# Use of Hg-Electroplated-Pt Ultramicroelectrode for Determining Elemental Sulphur in Naphtha Samples

**DOI:** 10.1155/2012/265687

**Published:** 2012-04-03

**Authors:** Carlos Eduardo de Andrade, Flávia C. de Souza, Daniella R. Fernandes, Sérgio A. S. Machado, Eliane D'Elia

**Affiliations:** ^1^Departamento de Química Inorgânica, Instituto de Química, Centro de Tecnologia, UFRJ, Avenida Athos da Silveira Ramos 149, Bloco A, Laboratório 634A, Cidade Universitária, 21941-909 Rio de Janeiro, RJ, Brazil; ^2^Instituto de Química de São Carlos, Universidade de São Paulo, Avenida do Trabalhador Saocarlense 400, 13566-590 São Carlos, SP, Brazil

## Abstract

This paper describes the applicability of a Hg-electroplated-Pt ultramicroelectrode in the quantification of elemental sulphur in naphtha samples by square-wave voltammetry. A reproducible deposition methodology was studied and is reported in this paper. This methodology is innovative and relies on the quality of the mercury stock solution to obtain reproducible surfaces required for the analytical methodology. All analyses were performed using a Hg-electroplated-Pt ultramicroelectrode (Hg-Pt UME) due to the low sensibility of such devices to ohmic drops in resistive solutions. The responses of the peak areas in voltammetric experiments were linear in all of the range studied. The method developed here is accurate and reproducible, with a detection limit of 0.010 mg L^−1^ and a good recovery range for both standard solutions of elemental sulphur (85 to 99%) and real naphtha sample (79%). These results attest to the potential for the application of this electroanalytical methodology in determining elemental sulphur in naphtha samples containing mercaptans and disulphides.

## 1. Introduction

Sulphur compounds have been studied intensively for many years due to the importance of the element in chemical, biological, and industrial areas. A keystone problem is related to the determination of sulphur and its compounds in drugs, in natural products, and in petroleum derivatives [1–7]. Sulphur is found in petroleum in several forms. In light fractions, the sulphur species are elemental sulphur, hydrogen sulphide, mercaptans, and disulphides, with the acid mercaptan forms dominating [8]. The complexity of these sulphur compound mixtures depends not only on the origin of the petroleum but also on the refining process [9].

 Sulphur causes corrosion and damages refining catalysts, decreasing the quality of the final product. Even in low concentrations, it is capable of catalysing the formation of other sulphur species in petroleum [9, 10]. The limit for total sulphur allowed in Brazilian naphtha samples is 500 ppm. Hydrogen sulphide, elemental sulphur, mercaptans and disulphides account for approximately 1% of the total sulphur content in naphthas. Therefore, developing methods for determining trace amounts of elemental sulphur have been an important challenge in chemical analysis [6].

 The most common methods found in literature are concerned with the quantification of total sulphur [5] and sulphide compounds [1, 10]. Although elemental sulphur has been studied for many decades [11], few changes can be observed from the different methodologies developed over time. Different working mercury electrodes, electrolyte solutions, and techniques have been combined to achieve better analytical conditions, as well as faster and more accurate analysis [6, 7, 12–21].

Recently, an electrochemical method was developed in which a small amount of sample was directly analysed by square-wave voltammetry (SWV) using a hanging mercury drop electrode [6, 7] in an electrochemical cell containing 10.0 mL of the supporting electrolyte, that is, a buffer solution containing 2% (v/v) glacial acetic acid and 1.4 mol L^−1^ sodium acetate in methanol. This method showed a linear response in the concentration range studied (0.010 to 0.238 mg L^−1^), low detection limit (0.003 mg L^−1^), and good recovery and precision.

 The chemical modification of electrode surfaces to carry out electrochemical analysis has several advantages in terms of selectivity, sensitivity, and efficiency for the determination of a species using electroanalytical techniques, such as metals analysis in different matrices using mercury film electrodes [22–31].

 Ultramicroelectrodes (UMEs) have received increasing attention for both kinetic studies of electrode processes and quantitative analyses due to their unique properties. Some of them include the low currents employed in electrochemical experiments, which result in negligible ohmic drops and fast responses due to small electrode capacitance. Another advantage is that the electrodes reach steady-state conditions in short times. In principle, these properties allow microelectrodes to be employed directly in resistive media without the addition of supporting electrolytes [27, 29–37].

 The objective of this work was to develop a new electroanalytical method to quantify elemental sulphur in Brazilian naphtha due to the need for mercury utilisation; this proposed methodology employs UME covered by a Hg film, thus minimising the utilisation of such a hazardous substance.

## 2. Experimental

All reagents were of pure grade and thus were used without any further purification, except for metallic copper powder, which was passed through an activation process [6, 7]. Methanol, ethanol, acetone, diethyl ether, 70% (m/m) nitric acid, 98% (m/m) sulphuric acid, 99% (m/m) glacial acetic acid, sodium acetate, potassium nitrate, mercury (I) nitrate, n-heptane, 1-butanethyol, 1-propanethyol, 2-methyl-2-propanethyol, dipropyl disulphide, diphenyl disulphide, elemental sulphur monoclinic, and metallic copper powder (particles <63 *μ*m and >230 mesh) were purchased from Merck. All sulphur standards were stored at temperatures between 10 and 15°C.

Voltammetric experiments were carried out at 25°C in a two-electrode cell with 30 mL of capacity placed in a Faraday cage to avoid electrical noises. The electrochemical cell presents a Teflon cover, which hosts both Hg-Pt-UME (mercury-platinum-ultramicroelectrode) as the working electrode and Ag/AgCl/KCl (3 mol L^−1^) as the reference electrode, as well as degassing facilities. The support-electrolyte mixture was a buffer solution containing 2% (v/v) glacial acetic acid and 1.4 mol L^−1^ sodium acetate in methanol.

All measurements were performed using an Autolab potentiostat, model PGSTAT 100, from Ecochemie with a current amplifier module controlled by the software program GPES 4.8. Square-wave voltammetry (SWV) was used to determine the elemental sulphur. The specific parameters used in all analyses were a 15 mV potential step, 50 mV pulse amplitude, 150 mV s^−1^ scan rate, 10 Hz frequency, and potential range from −0.100 to −0.800 V versus Ag/AgCl. All of these parameters were optimised in previous experiments.

### 2.1. Construction of Hg-Pt-UME

The platinum ultramicroelectrodes (Pt-UME) were constructed by embedding a 25 *μ*m diameter Pt wire (Goodfellow) in a Pyrex glass tube with a 0.50 mm internal diameter. The tips of the Pt ultramicroelectrodes were polished with emery paper until a metal microdisc was exposed at the surface. After this procedure, the microelectrodes were cleaned with purified water prior to use. The voltammetric characterisation consisted of analysing the electrochemical response of the Pt ultramicroelectrode in an electrolyte containing 1.0 × 10^−3^ mol L^−1^ of potassium hexacyanoferrate (III) in 0.10 mol L^−1^ KCl with a pH of 3. The voltammograms obtained exhibited the well-known sigmoid profile, which is characteristic of an electrochemical process controlled by spherical diffusion mass transport, as expected when using ultramicroelectrodes.

The Hg-Pt-UME preparation involves four steps: mechanical polishing, conditioning, characterisation, and mercury film electroplating on Pt-UME surface. It is important to perform mechanical polishing and conditioning to clean and guarantee a homogeneous platinum surface for electroplating. Polishing was performed using four different grids emery papers (400, 600, 1200, and 2000 papers). After polishing, the electrode was washed with 10% (v/v) HNO_3_ solution and water.

The conditioning step was carried out by repetitive cyclic voltammetry within a potential window of −0.4 to 1.75 V versus Ag/AgCl range at a 0.5-V s^−1^ scan rate in a 0.5 mol L^−1^ H_2_SO_4 _ solution. The number of cycles depends on the amount of impurities adhered to the electrode. This characterisation was carried out by cyclic voltammetry, from −0.2 to 1.45 V versus Ag/AgCl at 0.1 V s^−1^ in 0.5 mol L^−1^ H_2_SO_4_.

The mercury-plated ultramicroelectrode (Hg-Pt UME) was prepared by electroplating Hg, from a mercurous-ion solution, on the surface of the UME by amperometry. A solution containing 0.01 mol L^−1^ of Hg_2_(NO_3_)_2_ was prepared and added to a supporting electrolyte, 0.1 mol L^−1^ KNO_3_ and 0.5% (v/v) HNO_3 _[37]. The Pt-UME was immersed in this mixture, which was kept over a continuous flow of nitrogen for 600 s to remove oxygen and polarised at −0.1 V versus Ag/AgCl over a preestablished time interval. The electroplated film was monitored by optical microscopy (Digital Microscope Hirox model KH 7700) to obtain a platinum surface fully covered with the mercury film.

### 2.2. Voltammetric Procedure

The calibration curve was constructed by adding an aliquot of 100 *μ*L of elemental sulphur standard solutions prepared in heptane (0.125, 0.250, 0.375, and 0.500 mg L^−1^) into an electrochemical cell containing 20 mL of the supporting electrolyte solution. These solutions were purged with nitrogen for 600 s before the analysis. The calibration curve was then constructed by plotting peak area *versus* elemental sulphur concentration. For synthetic and real naphtha samples, aliquots of 100 *μ*L were also analysed in 20 mL of the supporting electrolyte solution. The synthetic sample was created by diluting elemental sulphur in heptane.

Each measurement was obtained with a newly obtained mercury film to assure the best reproducibility and repeatability of the measurements. This procedure must be performed due to the great affinity of elemental sulphur for the mercury surface. The sulphur adsorption process passivates the mercury surface [7]. Here, we have chosen to reconstruct the electrode to evaluate the reproducibility of the construction methodology and consequently the measurements.

### 2.3. Analytical Performance

 The analytical performance of the voltammetric method for the quantitative determination of elemental sulphur in naphtha samples was carried out by following a few basic steps, such as determining the selectivity, linearity, detection and quantification limits, recovery, and precision. The precision of mercury electroplating in the Pt-UME was analysed while considering the repeatability and intermediate precision.

The linearity study was performed using standard elemental sulphur solutions that were analysed in four concentrations ranging from 0.125 to 0.500 mg L^−1^. The analysis was performed in triplicate for each concentration.

The detection limit was experimentally obtained and calculated. The experimental detection limit was obtained from the elemental sulphur current signal, which was at least three times the magnitude of the largest noise in the voltammogram. The detection limit was also determined according to the 3*σ*/*b* criterion, where *σ* is the standard deviation of the blank analysis and *b* is the angular coefficient of the calibration curve. The blank was 20 mL of the supporting electrolyte mixture with 100 *μ*L of heptane, which was analysed in triplicate.

The recovery was evaluated by analysing a standard solution of elemental sulphur in heptane (synthetic sample) and a real naphtha sample. For the analysis of the synthetic sample, standard elemental sulphur solutions in 0.100, 0.200, and 0.400-mg L^−1^ concentrations were added directly into the electrochemical cell containing the supporting electrolyte solution. The real naphtha sample was analysed in its pure state and fortified with 0.250 mg L^−1^ of elemental sulphur. All analyses were performed in triplicate.

The repeatability was determined from the results obtained on the same day, whereas the intermediate precision was evaluated by comparing the results obtained on different days on the same instrument.

## 3. Results and Discussion

### 3.1. Construction of Hg-Pt-UME

The electroplating methodology used to form a mercury film on the Pt-UME surface is extremely important because the obtained electrodeposits must exhibit identical characteristics to assure the reproducibility of the electrochemical responses during the reduction of elemental sulphur to sulphide. Such electrochemical signals (reduction currents) are fundamentally determined using the electroactive surface area.

A typical chronoamperometric curve was obtained for mercury-film electroplating at −0.1 V on the Pt-UME. In the first stage of deposition, a thin layer of intermetallic species (Pt_2_Hg) is formed. This stage is followed by the spontaneous formation of mercury nuclei mainly near the edge of the Pt surface, where current densities are higher. With time, the nuclei coalesce until a full hemisphere is formed. This coalescence alters the surface area of the electrode and leads to indentations in the current deposition curves [37].

**Figure 1:**
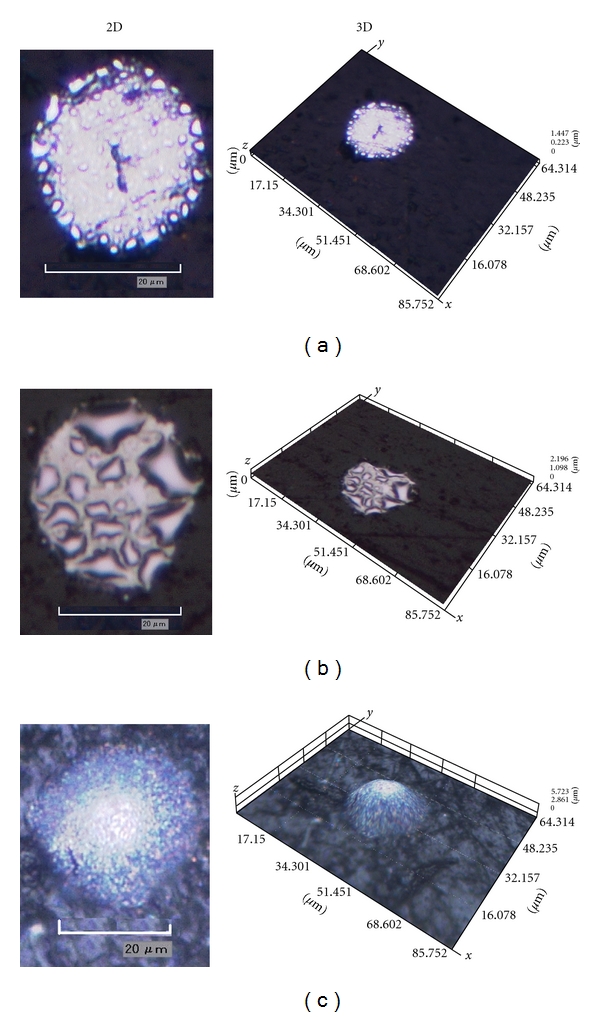
Photographs obtained via optical microscopy (magnified 2500 times) of the mercury film electroplating on the Pt-UME surface in mercurous solution at −0.1 V versus Ag/AgCl in 2D and 3D; deposition times were (a) 5 s, (b) 25 s, and (c) 300 s.

A study of deposition time was carried out to obtain a fully covered Hg-Pt-UME surface, which was evaluated by optical microscopy.  Figure 1 shows microphotographs of the mercury coating obtained at different deposition times, which agrees with the deposition steps discussed above. At short deposition times, the coating exhibits the morphological characteristics shown in Figure 1(a). The preferential formation of mercury nuclei occurs close to the platinum-glass interface; these nuclei coalesce (Figure 1(b)) over time to ultimately form a full hemisphere (Figure 1(c)). According to this Figure 1(c), the best deposition time was 300 s, when the platinum surface was fully covered with the mercury film.

**Figure 2:**
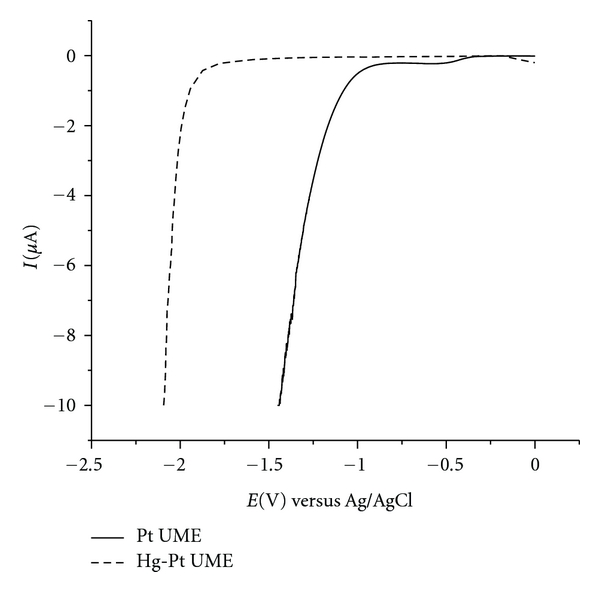
Current-potential curves for Pt and Hg-Pt UMEs in 0.1 mol L^−1^ KNO_3_.

The electrochemical behaviour of the Pt-UME and Hg-Pt-UME was evaluated by linear-sweep voltammetry in a 0.1 mol L^−1^ potassium nitrate aqueous solution (Figure 2). After mercury deposition, the onset of the water reduction wave shifted to more negative potentials by approximately −1.0 V, showing that the cathodic reaction becomes extremely slow on the Hg-Pt-UME due to the high hydrogen overpotential on mercury. Dirty or damaged electrodes can promote only a small shift in the overpotential of H_2_ evolution (approximately −0.20 V [37]). Clean UMEs with a thick mercury deposit, however, are well behaved, as shown in Figure 2.

### 3.2. Precision of the Ultramicroelectrode

A study of the repeatability and intermediate precision of the Hg-Pt-UME was carried out to determine the variations in the charges obtained in each deposit for *n* replicates. The mass of mercury of the electroplated film is proportional to the charge transferred during the electrolysis in potentiostatic mode over a fixed time interval.  Table 1 shows the average charge and its standard deviation obtained with different mercury stock solutions of the same concentration used on the same day (conditions 1 and 2) and using the same mercury stock solution used on different days (conditions 3 and 4). The mean values for the electrodeposition charge obtained with different stock solutions of the same concentration on the same day resulted in a standard deviation *F*_cal_ higher than *F*_crit_; this suggests that the charge values obtained from two different stock solutions are different, while the mean charge values obtained from the same stock solution on different days are equivalent. Thus, the errors committed in preparing different stock solutions seem to be important in determining the reproducibility of the coating if the electrodeposition is performed in chronoamperometric mode over a fixed time interval. 

### 3.3. Applicability of the Hg-Pt-UME in the Voltammetric Method

**Figure 3:**
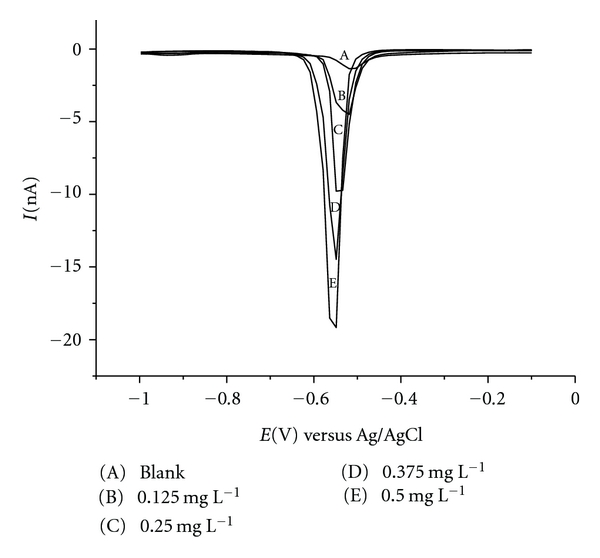
Square-wave voltammograms for different elemental sulphur concentrations in a buffer solution containing 2% (v/v) glacial acetic acid and 1.4 mol L^−1^ sodium acetate in methanol.

The electroactivity of elemental sulphur was investigated in a mixture of methanol, acetic acid, and sodium acetate by square-wave voltammetry with Hg-Pt-UME as the working electrode. The elemental sulphur was electroactive within a potential window of −0.50 to −0.67 V versus Ag/AgCl, with two electrons involved in the electrode process, and consequently, elemental sulphur is reduced to sulphydric acid [6, 7]. Therefore, the elemental sulphur concentration is proportional to the peak area of the voltammogram (Figure 3), and the calibration curve was acquired by fitting the data obtained with elemental sulphur standard samples (peak area (in nW) versus [*S*] (in mg L^−1^)) to the linear regression model. 

**Figure 4:**
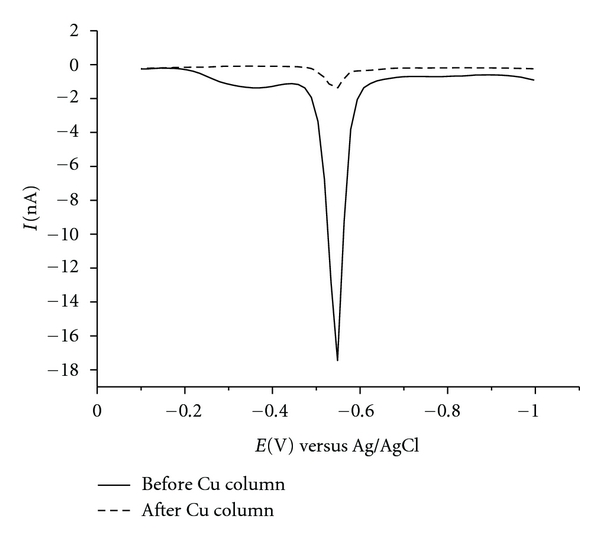
Voltammograms obtained with real naphtha sample before and after elution by the copper column.

From our recent studies [6, 7], disulphides were found to be the only possible interferents during elemental sulphur determination, even if the potential window is extended from −0.1 to −0.8 V versus Ag/AgCl. We found that mercaptan species are also electroactive but only within a different potential range, that is, between −0.3 and −0.50 V versus Ag/AgCl. To overcome the interference caused by disulphides, we used a copper powder column because mercaptans and elemental sulphur react with the copper forming cuprous mercaptides, while disulphides do not react [6, 7]. The elemental sulphur concentration can then be calculated by subtracting the area of the peak relative to the voltammetric curve after passing the sample through a copper column (due to the presence of disulphides) from the total peak of the real naphtha sample (which is obtained before passing the sample through a copper column) (Figure 4).

**Figure 5:**
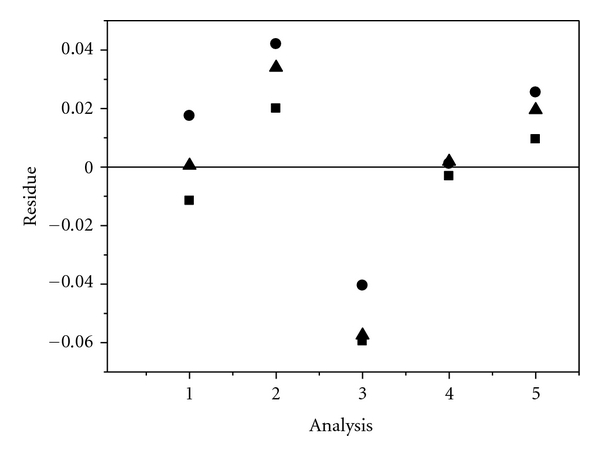
Residue graph obtained from the differences between the values calculated from the straight line of the calibration curve and the values obtained experimentally.

To evaluate the linearity of the method, standard elemental sulphur solutions were analysed in four concentrations ranging from 0.125 to 0.500 mg L^−1^.  Table 2 presents the experimental data shown in Figure 3 and RSD values for different concentrations of elemental sulphur. The Cochran test was applied, and the results (*C*_cal_ < *C*_crit_) show that the data are homoscedastic in the range studied. Moreover, according to the residue plot (Figure 5), the residues are randomly distributed around the zero line, and no pattern is observed. The proposed method is therefore adequate, and linear regression can be used. An *R* value of 0.9946 obtained was considered acceptable for this analysis.

For the proposed voltammetric method, the detection limit was experimentally obtained and calculated according to the IUPAC 3*σ*/*b* criteria, and these values were 0.010 and 0.027 mg L^−1^, respectively. Small concentrations of elemental sulphur could therefore be determined using this proposed methodology. However, the use of Hg-Pt-UME did not lead to a lower detection limit if compared with that one obtained using a hanging mercury drop electrode [6].

Table 3 shows the recovery results obtained from synthetic samples of different elemental sulphur concentration, whereas Table 4 shows the recovery from a real naphtha sample that was both pure and fortified with elemental sulphur. The voltammetric method presented good recovery values for both standard solutions and real samples. In the first case, the recovery range was 85 to 99%, whereas in the real sample, the recovery range decreased to 79%. These results suggest good performance, considering the recovery of the trace elemental sulphur. Moreover, there is an actual problem with the deposition of elemental sulphur in natural gas transmission line systems; in this case, an electrochemical method using Hg-Pt-UME could help in the sulphur analysis [38].

## 4. Conclusions

It has been shown that elemental sulphur can be adequately quantified in naphtha samples by an electroanalytical methodology using square-wave voltammetry and a Pt-UME modified by Hg coating.

In such an experimental setup, lower concentrations of elemental sulphur can be detected than those allowed by Brazilian regulations for naphtha samples (500 ppm) containing mercaptans or disulphides.

To this end, when coating with Hg, which was aimed to minimise the utilisation of such hazardous substances, Pt-UME was the best choice. Moreover, the enhanced mass transport towards the UME surfaces results in an enhanced analytical signal.

A new method to electroplate the Hg coating in a very reproducible manner is the most important contribution of this work. Previous studies have dealt with Hg static drop or even dropping mercury electrodes, using a much higher amount of Hg.

## Figures and Tables

**Table 1:** Charges obtained for mercury film electroplating under different conditions.

Condition	Charge average/*C*	*S*/*C*	*F*_cal_	*F*_crit_
(1) Solution A/day X	−1.25 × 10^−5^ (*n* = 12)	2.6 × 10^−7^	3.33	2.82
(2) Solution B/day X	−1.14 × 10^−5^ (*n* = 12)	1.4 × 10^−7^		
(3) Solution C/day X	−1.25 × 10^−5^ (*n* = 8)	2.7 × 10^−7^	1.26	3.79
(4) Solution C/day Y	−1.21 × 10^−5^ (*n* = 8)	3.0 × 10^−7^		

*S: standard deviation; *F*_cal_: calculated Snedecor *F* value; *F*_crit_: critical Snedecor *F* value; *n*: mercury film electroplating number.

**Table 2:** Linearity study of synthetic samples using elemental sulphur.

*S*_concentration_/mg L^−1^	blank	0.125	0.250	0.375	0.500
Area average/nW	0.137	0.368	0.485	0.739	0.959
RSD	11%	3.0%	2.2%	0.36%	0.84%
*C*_cal_	0.311	—	—		—
*C*_crit_	0.746	—	—		—

* All area values were considered with 95% confidence; RSD: relative standard deviation, *C*_cal_: calculated value of Cochran, and *C*_crit_: critical value of Cochran.

**Table 3:** Recovery results for synthetic samples of elemental sulphur.

Sample	*S*_added_/mg L^−1^	*S*_found_/mg L^−1^	Recovery/%
1	0.100	0.085 ± 0.031	85
2	0.200	0.198 ± 0.013	99
3	0.400	0.393 ± 0.011	98

**Table 4:** Recovery results for real naphtha sample, pure and fortified.

Sample	*S*_added_/mg L^−1^	Area_DBACC_/nW	*S*_found_/mg L^−1^	Recovery/%
1	—	7.15 × 10^−1^ ± 0.03	0.360 ± 0.065	—
2	0.250	9.09 × 10^−1^ ± 0.14	0.480 ± 0.003	79

*Area_DBACC_: difference of area value between elemental sulphur calculated before and after passing the sample through the copper column.
